# Spirituality and Palliative Care

**DOI:** 10.4103/0973-1075.76241

**Published:** 2011-01

**Authors:** Bert Broeckaert

**Affiliations:** Interdisciplinary Centre for the Study of Religion and Worldview, K.U. Leuven, Leuven, Belgium

**Keywords:** Spirituality, Religion, Palliative care, Ethics

## Abstract

This paper shows how palliative care developed as a reaction to the compartimentalized technical approach of modern medicine. But what does it mean if we say palliative care wants to treat patients as whole persons? A few pitfalls need to avoided. All disciplines involved in palliative care should act within the limits of their own specific professional role. Physicians and nurses should certainly not force patients into spiritual or religious discussions or practices. They should understand that religion and spirituality also influence the ethical (and thus medical) choices people make, respect their own conscience and worldview too and cultivate conscious compassion.

At the start of modern medicine, the ancient holistic paradigm of healthcare that was present in many cultures gradually became replaced by a dualistic approach that separated cure for the body and care for the soul. Indeed, Hippocratic medicine started from a causal and technical approach to body and illness. Thus, in western culture, the holistic priests of traditional healthcare were replaced by two distinct figures: the Christian priests became the healers of the soul, the Hippocratic doctors the healers of the body. It is important to see that although traditional medicine too contains lots of elements of sometimes very specialized empirical knowledge, western medicine has been and continues to be very successful-and this worldwide-precisely because it freed itself from a prescientific mythical understanding of man. By using a compartmentalized technical approach, a rational and analytical method, an empirically orientated scientific method that continuously integrates an ever-growing and ever-adapting body of scientific evidence and knowledge, enormous progress has been made.

However, notwithstanding this triumph of western medicine with its antibiotics, organ transplants, blood transfusions, sophisticated surgical interventions, and curative successes in numerous cancer patients, at the same time something went wrong. Ironically, the specialized and technical approach that caused our great stride forwards is contested today by many people as a problematic, one-sided, and even inhumane model. Patients want to be approached as a person who is suffering, not as a faceless individual with bodily pain or a dehumanized diseased or malfunctioning organism.

Especially when confronted with its own limits, when all curative or life-lengthening attempts have been in vain, western medicine shows its shortcomings. In many countries, palliative care developed precisely as a reaction to the incapacity of traditional western medicine to take human suffering at the end of life seriously. Without at the same time neglecting the need for specific and sometimes quite technical approaches to the symptoms terminally ill patients are suffering from, from the start palliative care was much more, much broader than just technical palliative medicine. By opting for a multidisciplinary or rather interdisciplinary approach, palliative care has put and puts the person of the patient (with his/her physical, psychological, social, and existential/spiritual dimensions) at the centre. By talking about suffering or “total pain” (Cicely Saunders), it makes clear that it understands very well that a terminal illness and the pain and symptoms it entails affect the whole person, shake the foundations of his or her existence. Palliative patients can suffer terribly even when they have no severe physical symptoms or when their physical symptoms are well controlled. Coping with this existential suffering, with this “cry for meaning” (Victor Frankl) may sometimes be more important than medical or technical solutions. Indeed, the existential or spiritual domain is an important determinant of quality of life for many patients.

The fact that in western palliative care we tend to speak about the existential or spiritual dimension, about spirituality and spiritual care and not so much about religion, the fact that even the word spirituality for some people is an unacceptable one, is of course not a simple coincidence. It is the result of specific historical developments in western society, the result of what sociologists have called secularization, i.e., the process by which religion, in casu Christianity, gradually lost its impact on different aspects and institutions of western society. If we nowadays and especially in a western context want to talk about the existential needs of people, the term religion has indeed become too narrow, as a lot of people in the west would not call themselves religious or do not adhere anymore to a specific religion. Even the term spirituality can sound too religious, too Christian for some. However, talking about spirituality rather than about religion is not just choosing a broader, more encompassing term. The stress on individual spirituality rather than on religion (which seems to focus more on the external, institutional, and group dimension) fits very well with the general idea of a secularized western society in which personal autonomy is stressed and religion, worldview, spirituality have become privatized and individualized in an important way. However, this privatization of religion and worldview is in an Important way a typically western phenomenon. This we should keep in mind when talking about religion and spirituality in the lives of people of non-western societies or of minorities of non-western descent, where the community aspect of religion is often quite important. It is clear that the role of religion and religions in, for instance, Indian society is quite different from the role religion plays in Belgium or The Netherlands.

But what do we mean then if we say that we want to treat our patients as whole persons and take their fundamental existential, spiritual, or religious needs seriously? How to Cope-as palliative care physicians, nurses, social workers, psychologists-with existential suffering? Here, we need to clarify our objectives: everybody will agree that in palliative care and probably in all care “sensitivity” for the patient’s spiritual need is necessary. However, this sensitivity is of course something else than the “integration” of religious or spiritual beliefs into for instance medical practice. We may be in search of a new holistic approach, but we have nothing to gain by going back to the prescientific holistic approach of the pre-Hippocratic tradition. Therefore, I think, some pitfalls need to be avoided.

1. In the care for terminally ill patients we–that is physicians, nurses, social workers, psychologists,… – all have a role to play, and all of us, within the limits of that *specific professional role* that is ours, have to look to the patient as a person, as a “whole” person. Physicians and nurses should be sensitive to all aspects of the patient’s experience, including the existential and spiritual. However, as far as spiritual care is concerned, their role is quite different from the role played by chaplains, pastoral workers, spiritual counselors, or other professionals in the field of spiritual care. I agree with Sloan *et al.* that “when doctors depart from areas of established expertise to promote a non-medical agenda, abuse their status as professionals.” There is indeed “an important difference between ‘taking into account’ […] religious [or spiritual] factors and ‘taking them on’ as the objects of interventions.”[1] Taking them on is not the task of the physician or the nurse. Physicians and nurses should be sensitive to the spiritual needs of people, but they have to operate within their own professional limits. The professional expertise of pastoral workers and spiritual counselors (that can pray with patients, perform rituals, listen to their existential questions, reconstruct their life stories, …) is needed and is an important ingredient of the *interdisciplinary* and encompassing approach that palliative care stands for.
2. *Avoid to be a missionary:* Given the specific professional role of the physician and the nurse, given the fact that spirituality has a very personal dimension, given the fact that most societies are of having become very pluralistic in this field, physicians should certainly not force patients into spiritual or religious discussions or practices.
3. It is important to understand that the importance of religion, worldview and spirituality at the end of life is not just a matter of spiritual care or just a matter of spiritual counseling and religious rituals. Since 2001 our research group is particularly interested in the way religion and worldview influence ethical attitudes and practices at the end of life.[2] Whether we want it or not or realize it or not, our medical acts, what we do or do not at the end of life is not only the result of our technical knowledge and technical considerations, but is in an important way determined by our ethical beliefs and attitudes. These beliefs and attitudes are in an important way linked to our worldview, our spirituality, our religion. And this is both true for our patients, their families, and the communities they belong to, and for ourselves, professionals working in end of life care. Because of its *ethical and, thus, also medical impact,* it is mandatory for palliative care to take religion and spirituality seriously. What as a medical treatment is acceptable for patients or not, is in an important way determined by their worldview or religion.
4. *Avoid to be a chameleon:* Physicians and nurses should maintain integrity and should not say or do anything that violates their own spiritual or religious views, also with regard to the delicate treatment decisions that have to be taken at the end of life. An example of this issue may be given from my own country, Belgium, that since 2002 has a law regulating euthanasia. This law, which has broad political and popular support, has started transforming medical culture. In Belgium we have moved from a complete taboo about this issue to a situation in which many people believe that they have a right to euthanasia, which results in patients or sometimes even family demanding in an aggressive way euthanasia from their doctor. Thus, physicians are sometimes put in a defensive position that is not respected by patients. Even when it is important to put the patient at the centre, this can never mean that the physician or the caregiver is reduced to the position of a chameleon that just accommodates to the situation, thus giving up his own ethical and existential integrity. Physicians and nurses should also respect their own conscience, their own spirituality, or worldview.
5. *Cultivate conscious compassion:* be sensitive to the patient’s view and aware of your own view in order not to get defensive or offensive. The future of a “whole person”-approach in palliative care is a conscious compassion. For “whole person spiritual care” this may mean “compassion and prayer” or “compassion and rituals.” For “whole person palliative medicine” this may mean “compassion and morphine,” i.e., a combination of empathy and technical knowledge and interventions. Our common goal is the well-being of the patient. It means the healing of his life, not just of his body. It means promotion of happiness and reduction of suffering. And as Frankl has shown us, this means giving meaning to patient’s lives. It seems appropriate to end with a narrative, written down by Viktor Frankl:

*One week later she died. During the last week of her life she was no longer depressed but on the contrary proud and full of belief. Before that, she had lots of difficulties because she thought she was useless. Our talk made her aware that her life was meaningful and even her suffering made sense. The last words she spoke were: My life was not meaningless. My life is a monument.*[3]
